# Electronic fluctuation difference between trimethylamine *N*-oxide and *tert*-butyl alcohol in water

**DOI:** 10.1038/s41598-022-24049-0

**Published:** 2022-11-12

**Authors:** Nahoko Kuroki, Yukina Uchino, Tamon Funakura, Hirotoshi Mori

**Affiliations:** 1 Department of Applied Chemistry, Faculty of Science and Engineering, Chuo University, Bunkyo-ku, Tokyo, 112-8551 Japan; 2 JST, ACT-X, Kawaguchi, Saitama 332-0012 Japan; 3 Department of Chemistry and Biochemistry, Graduate School of Humanities and Sciences, Ochanomizu University, Bunkyo-ku, Tokyo, 112-8610 Japan; 4 Department of Theoretical and Computational Molecular Science, Institute for Molecular Science, Myodaiji, Okazaki, 444-8585 Japan

**Keywords:** Chemical physics, Biophysical chemistry, Computational chemistry, Molecular dynamics, Quantum chemistry

## Abstract

Although small organic molecules in cells have been considered important to control the functions of proteins, their electronic fluctuation and the intermolecular interaction, which is physicochemical origin of the molecular functions, under physiological conditions, i.e., dilute aqueous solutions (0.18 mol L^−1^), has never been clarified due to the lack of observation methods with both accuracy and efficiency. Herein, the time evolutions of the interactions in dilute aqueous trimethylamine *N*-oxide (TMAO) and *tert*-butyl alcohol (TBA) solutions were analyzed via ab initio molecular dynamics simulations accelerated with the fragment molecular theory. It has been known that TMAO and TBA have similar structures, but opposite physiological functions to stabilize and destabilize proteins. It was clarified that TMAO induced stable polarization and charge-transfer interactions with water molecules near the hydrophilic group, and water molecules were caught even near the CH_3_– group. Those should affect protein stabilization. Understanding the solution dynamics will contribute to artificial chaperone design in next generation medicine.

## Introduction

Small organic solutes in cells have various effects on proteins. For example, trimethylamine *N*-oxide (TMAO, Fig. S1a), which consists of N^+^O^−^ and methyl (CH_3_–) groups, has been found in deep-sea fishes and is a known osmolyte that preserves the physiological functions of proteins^1^. The effects are often compared with the ions like (CH_3_)_4_ N^+^ and PO_4_^3−^ that are more capable of structuring water in the Hofmeister series^2^. However, the preservation mechanism of the osmotic pressure is still under debate; the proposed explanations include an attractive direct interaction between TMAO and proteins^2^ or indirect interactions via structural changes of an aqueous solution as a molecular aggregate^3,4^. If the mechanism by which TMAO allows physiological functional preservation in proteins is clarified, it would make fundamental scientific contributions to, for example, the next generation of medicine by accelerating the development of artificial chaperones and understanding the mechanism of atherosclerosis.

The biophysical chemistry and solution dynamics of aqueous TMAO solutions have been extensively investigated from experimental^5–14^ viewpoints. Vibrational and nuclear magnetic resonance spectroscopy indicated that both the N^+^O^−^ and CH_3_– groups of TMAO slow the dynamics of water molecules in a solution and that the N^+^O^−^ groups have a notable ability to capture water molecules^5–7,10,11,13^. These results have been carefully discussed by molecular dynamics simulations based on classical force fields^3,15–18^. However, it is well known that the results depend on the choice of the force field. Thus, ab initio molecular dynamics (AIMD) simulations are essential for explaining the experimental results precisely and designing the osmotic pressure regulation ability artificially. Although there have been several sub-picosecond-order Born–Oppenheimer AIMD simulations with the density functional theory^19–24^, previous studies have not revealed the intermolecular interaction, which is physicochemical origin of the molecular functions. Additionally, due to the high computational costs of the previous AIMD simulations, the target systems were limited within the small sizes, and the concentrations (over 0.5 mol L^−1^) were much higher than natural conditions (i.e., those in deep-sea fishes).

The object of this study is to clarify the electronic fluctuation and the temporal evolution of intermolecular interactions in TMAO aqueous solution with a realistic condition of concentration. For this purpose, we focused on solute–solvent interactions and applied the ab initio effective fragment potential-molecular dynamics simulation (EFP-MD), which is particularly suited to performing nanosecond-order AIMD simulations for systems containing several thousand atoms^25–30^, for dilute aqueous TMAO solutions (0.18 mol L^−1^). For comparison, a dilute aqueous solution of *tert*-butyl alcohol (TBA, Fig. S1b), which is known as a protein denaturant^5,6,16^, was also investigated.

## Computational method

The structures of TMAO, TBA, and H_2_O molecules in the gas phase were optimized using the Gaussian16 quantum chemistry program package^31^. The MP2/aug-cc-pVTZ^32^ level of theory was applied to the calculations, and the natural bond orbital (NBO) analysis was performed. The T_1_ diagnostic values^33^ of TMAO, TBA, and H_2_O molecules were 0.013, 0.010, and 0.010, respectively, confirming that there was no multireference nature. Using the wavefunctions for the optimized molecules, the EFPs at the aug-cc-pVTZ basis function were uniquely defined by the “MAKEFP” module implemented in the GAMESS-US program package^34^.

Before performing the EFP-MD simulations, we evaluated the accuracy of the EFPs. For this purpose, we decomposed the total interaction energies obtained by the quantum chemistry calculations (MP2/aug-cc-pVTZ) into electrostatic (*E*^ES^), exchange-repulsion (*E*^EXREP^), polarization with charge-transfer (*E*^POL^ + *E*^CT^), and dispersion (*E*^DISP^) interaction energy components through localized molecular orbital energy decomposition analysis (LMO-EDA)^35^ and compared them with the EFP results. In the LMO-EDA calculations, we applied the counterpoise method to correct the basis set superposition errors.

Subsequently, we performed a set of EFP-MD simulations for dilute aqueous TMAO or TBA solutions and pure water. In the EFP-MD simulations, we used a set of cubic periodic boxes with a side length of ~ 21 Å containing one solute molecule and 300 H_2_O molecules with a canonical (NVT) ensemble and a cutoff distance of 10 Å. Damping expressions were applied for long-range terms^25^. The simulation box size was defined to model the dilute aqueous solution (0.18 mol L^−1^), which was realistic concentration for deep-sea fishes. In the EFP-MD simulations, we used a time step of 1 fs and a temperature of 298.15 K (defined using a Nosé–Hoover thermostat). Under these conditions, a set of at least 0.8 ns equilibration and 2.5 ns production runs was performed to evaluate the self-diffusion constants, radial distribution functions (RDFs), and time-dependent intermolecular interaction energies.

## Results and discussion

The chemical accuracy of EFPs was verified for TMAO, TBA, and H_2_O. The EFPs reproduced dipole moments via high-precision ab initio quantum chemistry calculations within a 0.14 D error, which is more accurate than that of well-trained classical force field models^16,17^ (Table S1). The structural parameters of TMAO/TBA–H_2_O dimer models optimized by the EFPs agreed with the MP2 level of quantum chemistry calculation results within 0.14 Å (Figs. S2, S3, and Tables S2, S3). The slight difference in dimer formation validates the rigid rotor approximation in the EFP method, at least within our target systems. The total interaction energy and its components, calculated by the EFP method, near the stable conformation of the TMAO/TBA–H_2_O dimer, accurately reproduced the corresponding LMO-EDA at the MP2 level (Figs. S4, S5). The mean absolute error (MAE) of the total interaction energy obtained by EFP and MP2 was 2.0 kcal mol^−1^. The MAE of each interaction energy component (*E*^ES^, *E*^EXREP^, *E*^POL^ + *E*^CT^, and *E*^DISP^) was 1.0, 0.6, 1.9, and 0.6 kcal mol^−1^, respectively. The H_2_O–H_2_O interaction described by the EFP method has been established previously^29^. The chemical accuracy of the EFP method was thus confirmed. It should be noted that there is no cumbersome fitting in the definition of EFPs.

One of the ways to discuss the transport properties by MD simulations is calculating the self-diffusion coefficients using Einstein's equation (Eq. 1).

1 $$D=\underset{t\to \infty }{\mathrm{lim}}\frac{1}{6t}\langle {\left|{{\varvec{r}}}_{i}\left(t\right)-{{\varvec{r}}}_{i}\left(0\right)\right|}^{2}\rangle$$

The diffusion coefficient of water (*D*_water_) was experimentally observed to be 2.3 × 10^–9^ m^2^ s^−1^^36^, and it has been also reported that *D*_water_ in a dilute aqueous TMAO/TBA solution (~ 0.2 mol L^−1^) is ~ 10% lower than that in pure water^6,18,37^. Our nanosecond-order ab initio EFP-MD results successfully reproduced that these solutes slow the dynamics of water molecules (Table S4).

**Figure 1:**
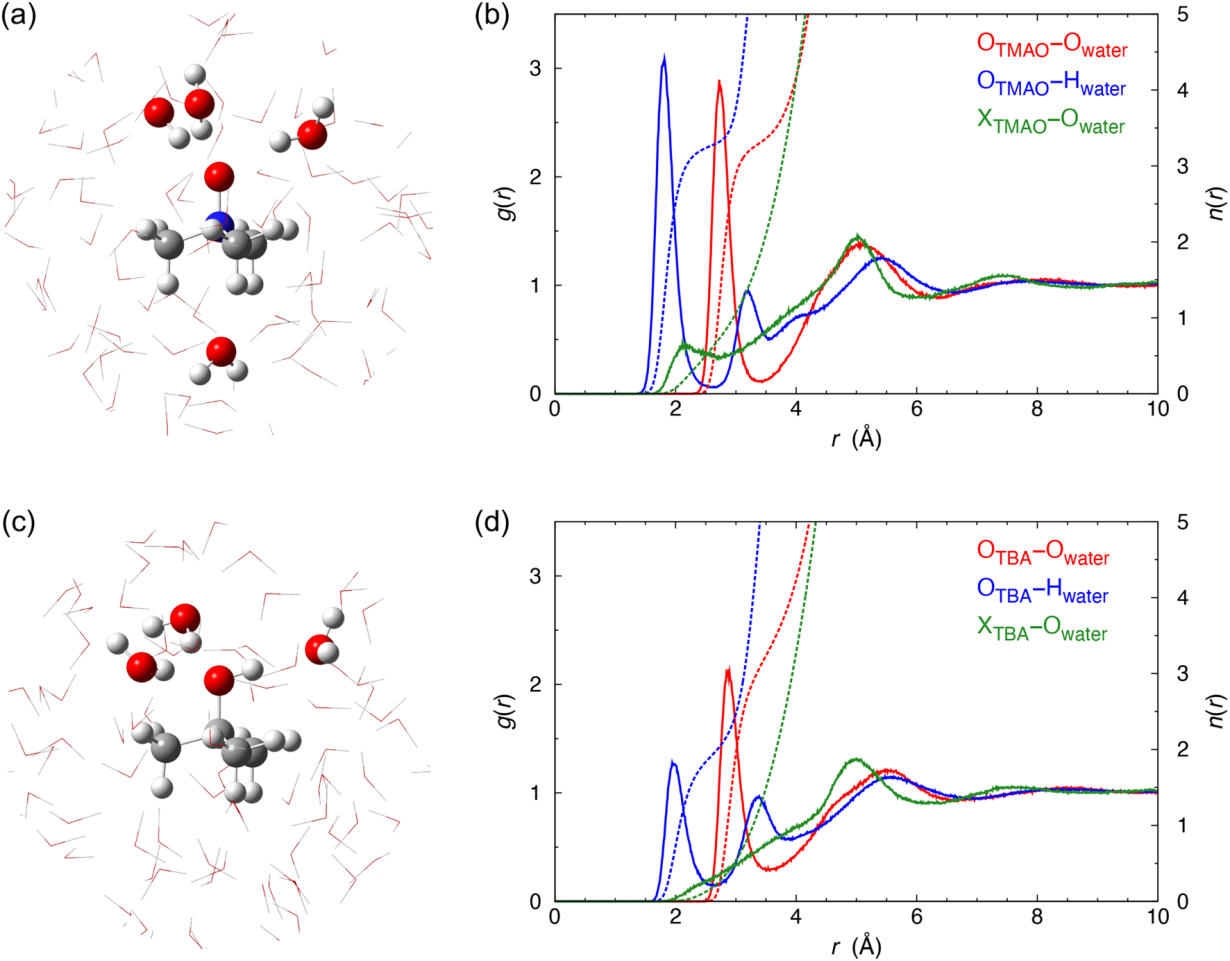
Solution structures simulated by EFP-MD. (**a**) Snapshot of aqueous TMAO. (**b**) Solute–solvent site RDFs, *g*(*r*), and hydration numbers, *n*(*r*), for aqueous TMAO. (**c**) Snapshot of aqueous TBA. (**d**) Solute–solvent site *g*(*r*) and *n*(*r*) for aqueous TBA. X_TMAO/TBA_ was defined as the center of mass of the three axial hydrogen atoms of the CH_3_– groups in TMAO/TBA.

Several solute–solvent site RDFs were calculated to investigate dilute aqueous TMAO/TBA solutions (Fig. 1 and Table S5). The coordination numbers of the top sites of solutes were evaluated by integrating the RDFs for O_TMAO/TBA_–O_water_ and O_TMAO/TBA_–H_water_ for the range up to the first minima. The coordination number calculated using O_TMAO/TBA_–O_water_ was 3.3 for both TMAO and TBA, while those calculated using O_TMAO/TBA_–H_water_ were 3.3, and 2.0 for TMAO and TBA, respectively. These results indicate that the hydrophilic groups of TMAO firmly trap three H_2_O molecules as hydrogen-bond donors, while those of TBA coordinate two H_2_O molecules and one H_2_O molecule as hydrogen-bond donors and acceptor, respectively. Focusing on the bottom sites of TMAO and TBA, i.e., the coordination numbers of X_TMAO/TBA_–O_water_, it is apparent that the CH_3_– groups of TMAO and TBA have different hydration properties. Therefore, the RDFs for X_TBA_–O_water_ have no peaks within 2 Å, while X_TMAO_–O_water_ has a coordination number of 0.7. The CH_3_– groups of TBA exhibit “hydrophobic hydration,” while those of TMAO proactively trap H_2_O molecules. The CH_3_– groups of TMAO and TBA enact differently in dilute solutions^5,18^.

The TMAO/TBA···H_2_O interaction correlation function ( *p*(*t*) )^38^ (Eq. 2) was calculated to clarify the effect of the N^+^O^−^, OH, and CH_3_– groups of each solute on the kinetics of the water molecules in the dilute aqueous TMAO/TBA solutions (Fig. S6).

2 $$p(t)=\frac{\langle h(0)h(t)\rangle }{\langle h(0)\rangle }$$

Here, *h*(*t*) is a step function defined as 1 when the distance between each solute and solvent site is smaller than the first minimum of each RDF (Table S5). Otherwise, *h*(*t*) is defined as 0. The TMAO/TBA···H_2_O interaction lifetimes (Table S6) were evaluated by fitting *p*(*t*) to $a{e}^{-t/{\tau }_{a}}+b{e}^{-t/{\tau }_{b}}\left(a+b=1\right)$ to the data in the range 0 < *t* < 100 ps (Fig. S6); the double exponential fitting was applied since it provided better results than those from the single exponential fitting.

The EFP-MD results indicated that the N^+^O^−^ group of TMAO and the OH group of TBA captured three H_2_O molecules with an average lifetime of 31.2 and 16.5 ps, respectively. The calculated lifetime for TMAO agreed with those obtained by dielectric spectroscopy (at least 50 ps at ~ 300 K)^13^ and previous AIMD simulations (30–50 ps at 320 K; for D_2_O solution)^19^. The lifetime near the CH_3_– group of TBA could not be defined due to the lack of a hydration shell (see Fig. 1), while that near TMAO was 6.9 ps. It was confirmed that the CH_3_– groups of TMAO could capture water.

**Figure 2:**
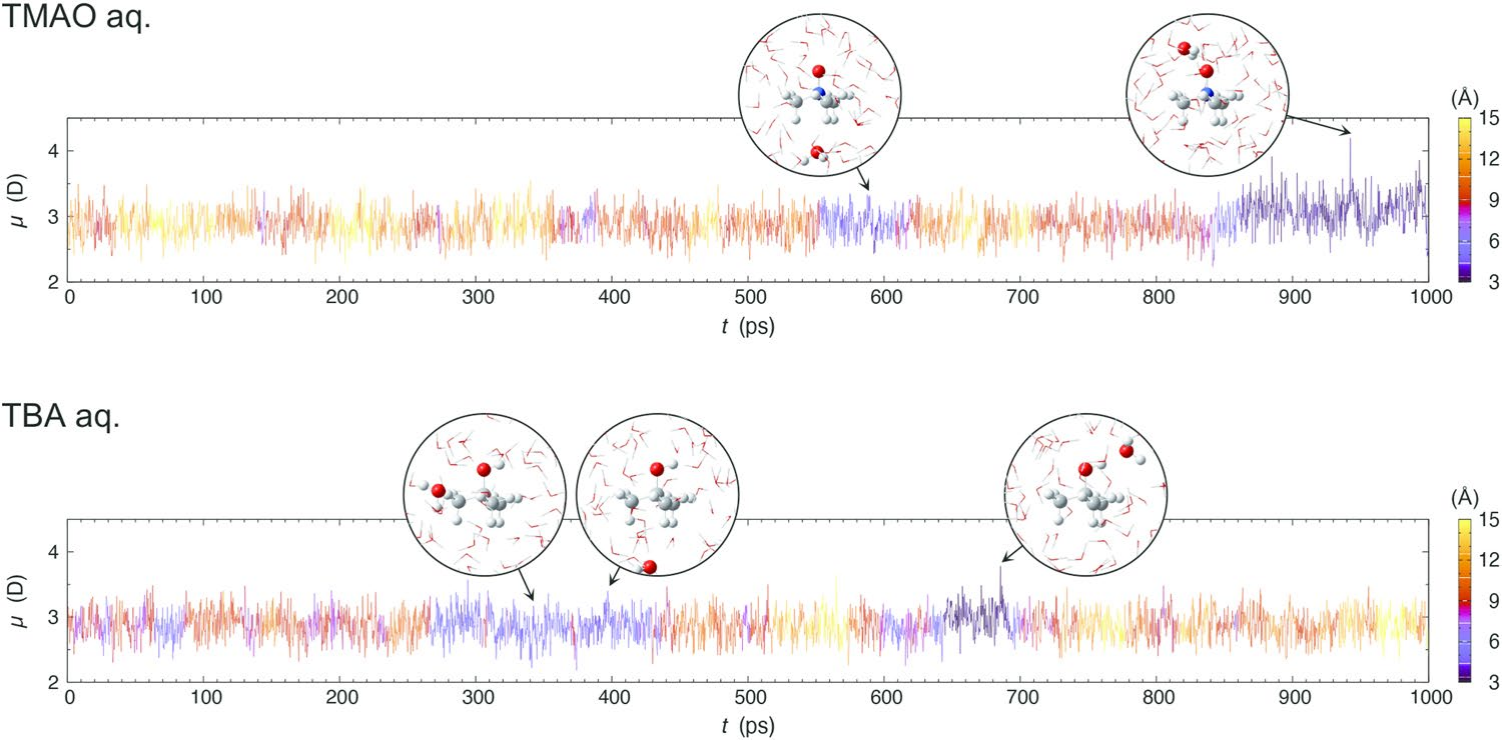
Temporal evolution examples of water dipole moments along 1 ns EFP-MD. The plot color represents the distances (N_TMAO_–O_water_/C_TBA_–O_water_) as indicated by the key on the right.

**Figure 3:**
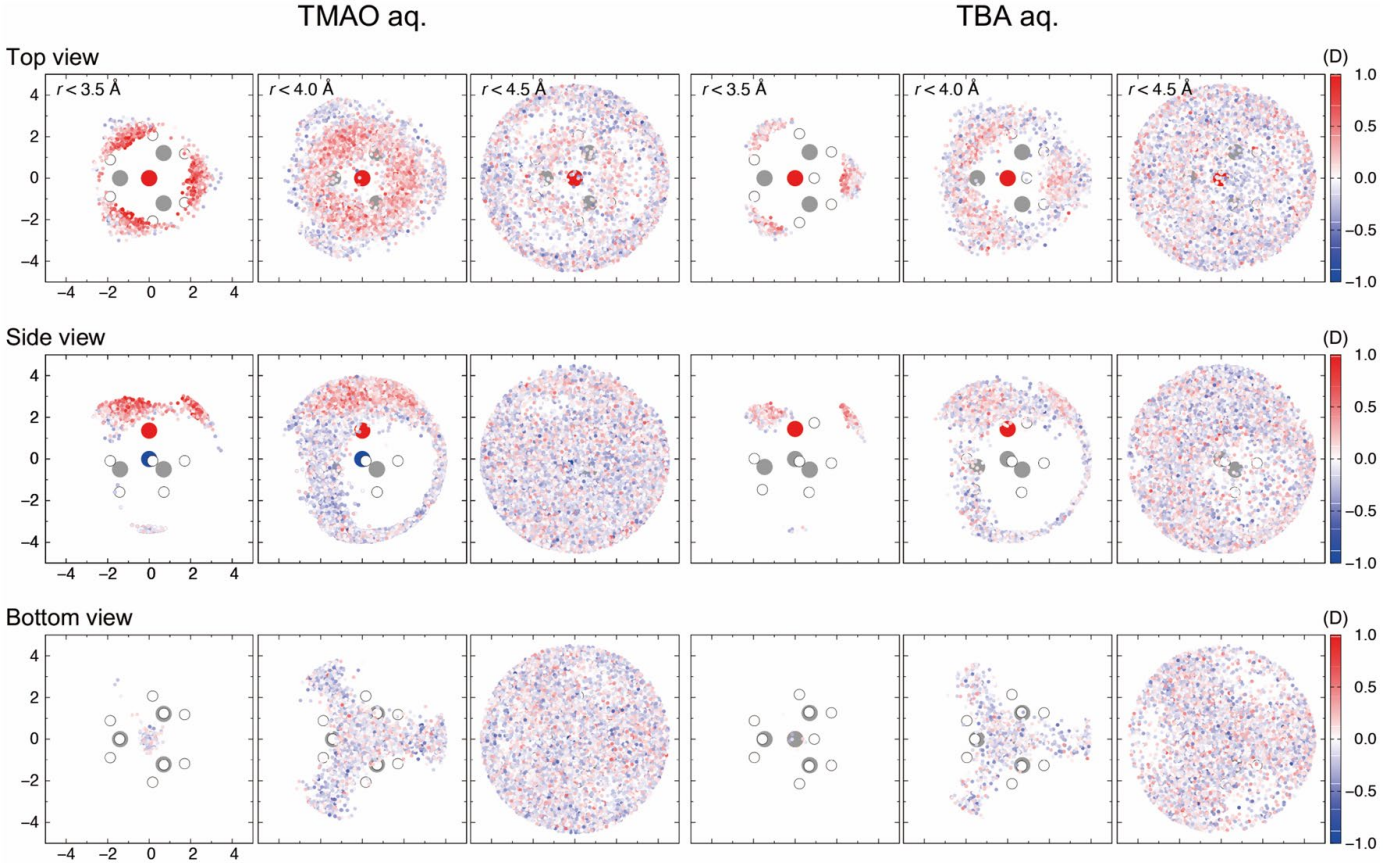
1 ns water fluctuation colored by the dipole moments of H_2_O molecules. The water molecules within 3.5, 4.0, and 4.5 Å from the solute are observed from the top, side, and bottom. The plot color represents the deviation from the pure water (2.87 D).

EFP-MD can be utilized to investigate the time evolution of dipole moments in dilute aqueous TMAO/TBA solutions. The dipole moment of the water molecule shown in Fig. 2 is enhanced (maximally 4.20 D) when it approaches the N^+^O^−^ group of TMAO. Similarly, when a water molecule approaches the OH group of TBA, the dipole moment is enhanced (maximally 3.78 D). The ensemble averages indicate that water molecules near the N^+^O^−^ group of TMAO (*r* < 3.5 Å) and the corresponding OH group of TBA increased the dipole moment by an average of 3.22 D (+ 12%) and 3.01 D (+ 5%), respectively, compared to the water molecules in pure water (Table S7). The former exhibits a more significant dipole moment because TMAO has a large dipole moment of 9.39 D in an aqueous solution. In general, molecules are stabilized by polarization in aggregated systems. Surprisingly, compared to the water molecules in pure water, the water molecules near the CH_3_– group of TMAO and TBA were found to have a decreased dipole moment (by −1% and −3%, respectively; Table S7). This is because the steric barrier of the CH_3_– group allows only a small number of water molecules to be coordinated around the waters with decreased dipole moments. The ensemble averages of the dipole moments indicate that the influence of the solute on water converges around 4.5 Å (Fig. 3 and Table S7).

**Figure 4:**
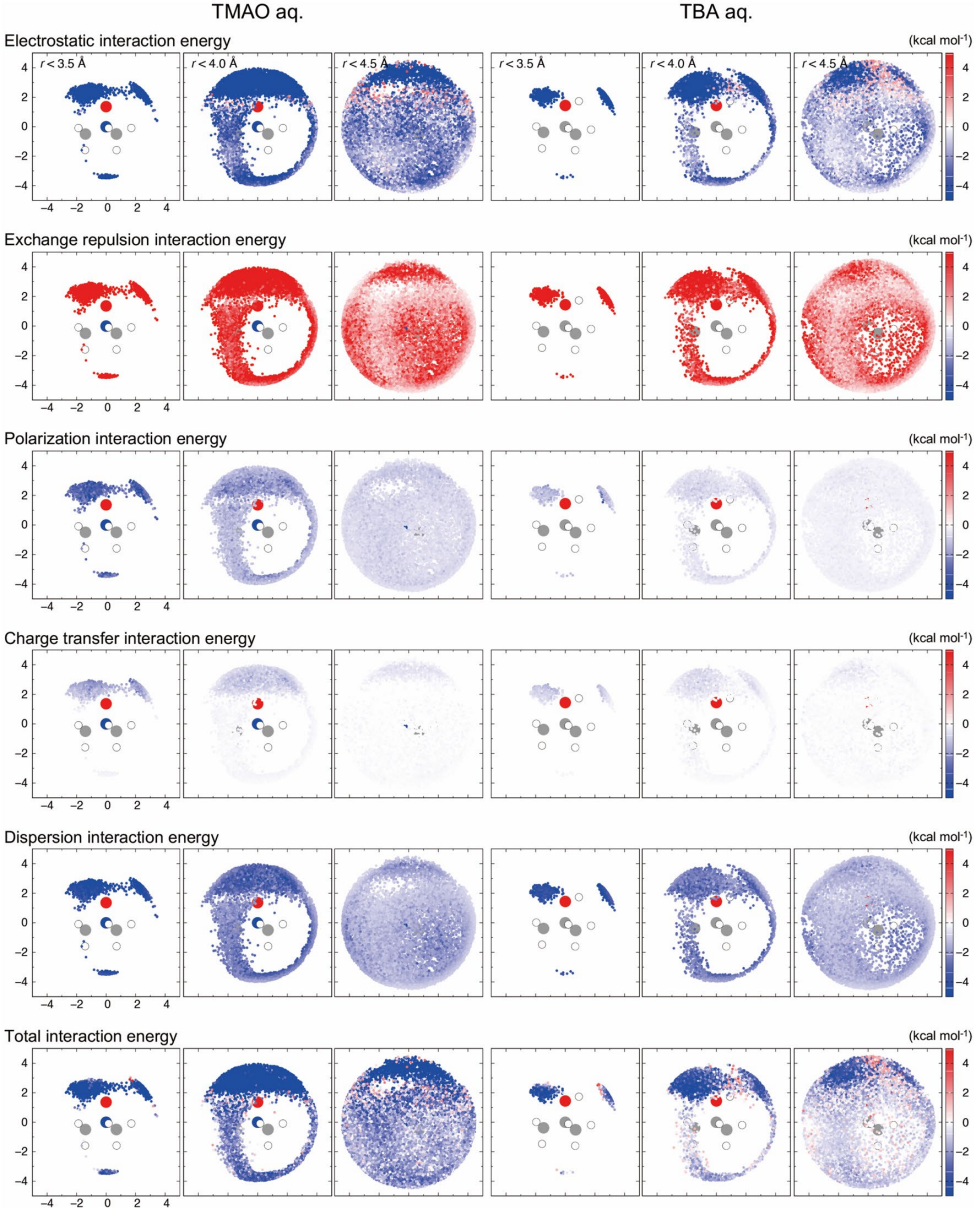
1 ns water fluctuation colored by TMAO/TBA–water interactions. The water molecules within 3.5, 4.0, and 4.5 Å from the solute are observed from the side.

The enhancing and diminishing of polarization on the surrounding water are considered to appear as differences in the interaction energy components (*E*^ES^, *E*^EXREP^, *E*^POL^, *E*^CT^, and *E*^DISP^) in the aqueous TMAO/TBA solution (Figs. 4, S7, S8, and Tables S8, S9). Therefore, the interaction energy components near the hydrophilic/hydrophobic groups are discussed.

First, the solute–solvent polarization and charge-transfer interactions in the vicinity of the N^+^O^−^ sites (*r* < 3.5 Å) in the TMAO solution were more than twice those corresponding to the OH sites in the TBA solution (Figs. 4, S7, and Table S8). The NBO analysis explains the charge-transfer interaction with a dimer model (Figs. S9, S10 and Table S10). The proton acceptor orbital of TMAO has a significant overlap integral with the H_2_O orbital around the hydrophilic group and facilitates the charge-transfer (0.04 e). However, the orbital overlap between the OH group of TBA and H_2_O is small; thus, the charge-transfer is small (0.01 e). Therefore, we can conclude that the factors that cause the N^+^O^−^ group to strongly supplement water in an aqueous TMAO solution are the polarization and charge-transfer interactions derived from the large polarization of TMAO.

Next, we analyzed the interaction between the CH_3_– group and the surrounding water molecules (*r* < 3.5 Å) in a TMAO/TBA solution. In this instance, the difference in the solute does not cause any difference in the charge-transfer and dispersion interactions (Figs. 4, S8, and Table S9). This can be explained by the small overlap between the proton donor orbitals of the CH_3_– groups of TMAO/TBA and the molecular orbitals of water (Figs. S9, S10, Table S10). However, the polarization interaction energy of TMAO is more than twice that of TBA. The large dipole moment of TMAO in an aqueous solution affects even the CH_3_– group. Assuming that the polarization interaction was zero, the interaction between the CH_3_– group of TMAO and water would stabilize at −0.8 kcal mol^−1^ because of the contribution of the dispersion interaction, which is similar to that of TBA, and a hydrophobic interaction would be induced. In conclusion, the attractive interactions near the CH_3_– group of TMAO are characterized by polarization interactions.

## Conclusions

This study represents an unprecedented attempt to discuss the influence of an osmolyte TMAO and a denaturant TBA on the electronic state fluctuation of dilute aqueous solutions by analyzing the time evolution of the intermolecular interactions; these interactions can be evaluated back to their physicochemical origin only via the ab initio EFP-MD method. The nanosecond-order EFP-MD method succeeded in reproducing the experimental results, i.e., TMAO and TBA slow the dynamics of water molecules. We analyzed the stabilizing effects of enthalpy, focusing on solute–solvent interactions. Our simulation results indicated that in dilute aqueous solutions, the dipole moment of the water molecules near the hydrophilic group of TMAO and TBA increased by an average of 12% and 5%, respectively. The dipole moment of the CH_3_– group decreased by an average of −1% and −3% for TMAO and TBA, respectively. When the chemical structures of the solutes were similar, the solute–solvent interaction characteristics changed depending on the local structure and polarity of the site. TMAO allowed stable polarization and charge-transfer interactions with water molecules near the hydrophilic group, and the large solute polarization affected water molecules near the CH_3_– group. However, the polarization of TBA was negligible and did not affect water molecules near the CH_3_– group; the interaction was hydrophobic. The effect of small amphiphilic molecules on the change in the electronic state in aqueous solutions is significant, and it will be necessary in the future to investigate the mechanism by which osmolytes and denaturants control the stability of proteins in biological environments using ab initio simulations taking electronic fluctuation effects into account.

## Supplementary Information

Supplementary Information.

## Data Availability

Data generated or analyzed during this study are included in the main article or Supplementary information.
