# Supplementary material for: Electronic fluctuation difference between trimethylamine N-oxide and tert-butyl alcohol in water
Source: Sci Rep. 2022 Nov 12;12:19417. doi: 10.1038/s41598-022-24049-0 (PMC9653398; doi:10.1038/s41598-022-24049-0)
Supplement: Supplementary file 1 — Supplementary Information. [file 41598_2022_24049_MOESM1_ESM.pdf]

Supplementary Information

Electronic fluctuation difference between trimethylamine *N*-oxide and *tert*-butyl alcohol in water

Nahoko Kuroki[1,2], Yukina Uchino[3], Tamon Funakura[1], and Hirotoshi Mori[1,4*]*

[1]Department of Applied Chemistry, Faculty of Science and Engineering, Chuo University; Bunkyo-ku, Tokyo, 112-8551, Japan.

[2]JST, ACT-X; Kawaguchi, Saitama, 332-0012, Japan.

[3]Department of Chemistry and Biochemistry, Graduate School of Humanities and Sciences, Ochanomizu University; Bunkyo-ku, Tokyo, 112-8610, Japan.

[4]Department of Theoretical and Computational Molecular Science, Institute for Molecular Science; Myodaiji, Okazaki, 444-8585, Japan.

* Corresponding authors:

kuroki.91d@g.chuo-u.ac.jp (N. Kuroki),
qc-forest.19d@g.chuo-u.ac.jp (H. Mori).

Supplementary Theory S3

Supplementary Figures and Tables

Chemical structures S8

Simulation resluts on monomers S9

Simulation resluts on dimers S10

Simulation resluts on dilute aqueous solution systems S17

Supplementary References S29

Supplementary Theory

Effective Fragment Potential (EFP)

In the EFP method,[1–6] under the rigid rotor approximation, intermolecular interactions in the target systems are described by multipoles and compact molecular orbitals for the constituent small monomer molecules (fragments) obtained by *ab initio* quantum chemistry calculations. If the fragment molecules are sufficiently small, the *ab initio* EFP-MD simulations can be performed with much lower computational costs than the conventional *ab initio* molecular dynamics (AIMD) methods based on the electronic structure theories such as the density functional theory.

The total inter-fragment interaction energy (E^{TOTAL}) is defined as the sum of the five energy components, that is, electrostatic (E^{ES}), exchange-repulsion (E^{EXREP}), polarization (E^{POL}), charge-transfer (E^{CT}), and dispersion (E^{DISP}) terms (Eq. S1).

$$E^{\mathrm{TOTAL}} = E^{\mathrm{ES}} + E^{\mathrm{EXREP}} + E^{\mathrm{POL}} + E^{\mathrm{CT}} + E^{\mathrm{DISP}} \tag{S1}$$

Hereinafter, $\{u, v\}$, $\{i, j, k, l\}$ and $\{I, J\}$ are the multipole expansion points, molecular orbitals and nuclei, respectively. $R_{\alpha\beta}$ is the distance between α and β. Superscripts $\{m, d, q, o\}$ and the subscripts $\{a, b, c\}$ represent the multipole orders (monopole, dipole, quadrupole, and octopole) and the *xyz* components, respectively. Subsequently, the five terms in Eq. S1 can be evaluated without using any empirical parameters as follows.

- **Electrostatic interaction term**

E^{ES} can be evaluated with multipoles placed on the atom centers and the bond midpoints. All the contributions from multipole–multipole ($E_{kl}^{\mathrm{multi-multi}}$), nuclei–multipole ($E_{Il}^{\mathrm{nuc-multi}}$), and nuclei–nuclei ($E_{IJ}^{\mathrm{nuc-nuc}}$) interactions have been calculated.[7,8] Sufficient high-order multipole expansion up to the octopole (Eq. S2) allows us to precisely describe the electrostatic potential using quantum mechanics.

$$\begin{aligned} E^{\mathrm{ES}} &= \sum_{u,v}\sum_{M} E_{uv}^{\mathrm{multi-multi}} + \sum_{I,v}\sum_{M} E_{Iv}^{\mathrm{multi-nuc}} + \sum_{I,J} E_{IJ}^{\mathrm{nuc-nuc}} \\ &\simeq \sum_{u,v}\left(E_{uv}^{\mathrm{m-m}} + E_{uv}^{\mathrm{m-d}} + E_{uv}^{\mathrm{m-q}} + E_{uv}^{\mathrm{m-o}} + E_{uv}^{\mathrm{d-d}} + E_{uv}^{\mathrm{d-q}} + E_{uv}^{\mathrm{q-q}}\right) \\ &\quad + \sum_{I,v}\left(E_{Iv}^{\mathrm{nuc-m}} + E_{Iv}^{\mathrm{nuc-d}} + E_{Iv}^{\mathrm{nuc-q}} + E_{Iv}^{\mathrm{nuc-o}}\right) + \sum_{I,J} E_{IJ}^{\mathrm{nuc-nuc}} \end{aligned} \tag{S2}$$

Terms $E_{kl}^{\text{multi-multi}}$, $E_{Il}^{\text{nuc-multi}}$, and $E_{IJ}^{\text{nuc-nuc}}$ in the Eq. S2 can be evaluated as follows.

$$E_{uv}^{\text{m-m}} = \frac{q^u q^v}{R_{uv}} \tag{S3}$$

$$E_{uv}^{\text{m-d}} = \frac{q^u \sum_a^{x,y,z} \mu_a^v a}{R_{uv}^3} \tag{S4}$$

$$E_{uv}^{\text{m-q}} = \frac{q^u \sum_a^{x,y,z} \sum_b^{x,y,z} \Theta_{ab}^v ab}{R_{uv}^5} \tag{S5}$$

$$E_{uv}^{\text{m-o}} = \frac{q^u \sum_a^{x,y,z} \sum_b^{x,y,z} \sum_c^{x,y,z} \Omega_{abc}^v abc}{R_{uv}^7} \tag{S6}$$

$$E_{uv}^{\text{d-d}} = \frac{\sum_a^{x,y,z} \mu_a^u \mu_a^v}{R_{uv}^3} - 3\frac{\sum_a^{x,y,z} \sum_b^{x,y,z} \mu_a^u \mu_b^v ab}{R_{uv}^5} \tag{S7}$$

$$E_{uv}^{\text{d-q}} = -2\frac{\sum_a^{x,y,z} \sum_b^{x,y,z} \Theta_{ab}^u \mu_a^v b}{R_{uv}^5} + 5\frac{\sum_a^{x,y,z} \sum_b^{x,y,z} \Theta_{ab}^u ab \sum_c^{x,y,z} \mu_c^v c}{R_{uv}^7} \tag{S8}$$

$$E_{uv}^{\text{q-q}} = 2\frac{\sum_a^{x,y,z} \sum_b^{x,y,z} \Theta_{ab}^u \Theta_{ab}^v}{3R_{uv}^5} - 20\frac{\sum_a^{x,y,z} \sum_b^{x,y,z} \Theta_{ab}^u b \sum_c^{x,y,z} \Theta_{ac}^v c}{3R_{uv}^7} + 35\frac{\sum_a^{x,y,z} \sum_b^{x,y,z} \Theta_{ab}^u ab}{3R_{uv}^9} \tag{S9}$$

$$E_{Iv}^{\text{nuc-m}} = \frac{Z^I q^v}{R_{Iv}} \tag{S10}$$

$$E_{Iv}^{\text{nuc-d}} = \frac{Z^I \sum_a^{x,y,z} \mu_a^v a}{R_{Iv}^3} \tag{S11}$$

$$E_{Iv}^{\text{nuc-q}} = \frac{Z^I \sum_a^{x,y,z} \sum_b^{x,y,z} \Theta_{ab}^v ab}{R_{Iv}^5} \tag{S12}$$

$$E_{Iv}^{\text{nuc-o}} = \frac{Z^I \sum_a^{x,y,z} \sum_b^{x,y,z} \sum_c^{x,y,z} \Omega_{abc}^v abc}{R_{Iv}^7} \tag{S13}$$

$$E_{IJ}^{\text{nuc-nuc}} = \frac{Z^I Z^J}{R_{IJ}} \tag{S14}$$

$\{q, \mu, \Theta, \Omega\}$ are the multipoles (monopole, dipole, quadrupole, and octopole) evaluated by Stone's distributed multipole analysis for the *ab initio* wave functions of the small fragment molecules.[7]

- **Exchange-repulsion interaction term**

E^{EXREP} can be extracted from the Heitler-London energy by subtracting the classical Coulomb term and the energies of the fragment molecules ($\mathrm{M_1}$ and $\mathrm{M_2}$) (Eq. S15).

$$E^{\mathrm{EXREP}} = \frac{\langle \Psi_{\mathrm{M_1}}\Psi_{\mathrm{M_2}} | \hat{A}\hat{H}_{\mathrm{M_1M_2}} | \Psi_{\mathrm{M_1}}\Psi_{\mathrm{M_2}} \rangle}{\langle \Psi_{\mathrm{M_1}}\Psi_{\mathrm{M_2}} | \hat{A}\Psi_{\mathrm{M_1}}\Psi_{\mathrm{M_2}} \rangle} - \langle \Psi_{\mathrm{M_1}}\Psi_{\mathrm{M_1}} | \hat{V}_{\mathrm{M_1M_2}} | \Psi_{\mathrm{M_2}}\Psi_{\mathrm{M_2}} \rangle - E_{\mathrm{M_1}} - E_{\mathrm{M_2}} \tag{S15}$$

Here, $\hat{H}_{\mathrm{M_1M_2}}$ is the Hamiltonian for the supermolecule ($\mathrm{M_1}$– $\mathrm{M_2}$), given by the individual Hamiltonians for the fragment molecules ($\mathrm{M_1}$ and $\mathrm{M_2}$) plus the interaction operator ($\hat{V}_{\mathrm{M_1M_2}}$). $\hat{A}$ is an antisymmetrizer, given by Eq. S16.

$$\hat{A} = P_0 - P_1 + P_2 - P_3 + \cdots \tag{S16}$$

P_n is the n-th order permutation operator. Truncating $\hat{A}$ at P_1 and expanding Eq. S16 using the localized molecular orbitals (LMOs) for fragments $\mathrm{M_1}$ and $\mathrm{M_2}$, the following equation is derived (Eq. S17).

$$E^{\mathrm{EXREP}} \simeq \sum_{k,l} \left\{ \begin{array}{l} -4\sqrt{\dfrac{-2\ln|S_{kl}|}{\pi}}\dfrac{{S_{kl}}^2}{R_{kl}} - 2S_{kl}\left(\displaystyle\sum_{i\in \mathrm{M_1}} F_{ki}^{\mathrm{M_1}} S_{il} + \sum_{j\in \mathrm{M_2}} F_{lj}^{\mathrm{M_2}} S_{jk} - 2T_{kl} \right) \\ +2{S_{kl}}^2\left(-\displaystyle\sum_{J\in \mathrm{M_2}} \frac{Z_J}{R_{kJ}} - \sum_{I\in \mathrm{M_1}} \frac{Z_I}{R_{lI}} + 2\sum_{j\in \mathrm{M_2}} \frac{1}{R_{kj}} + 2\sum_{i\in \mathrm{M_1}} \frac{1}{R_{li}} - \frac{1}{R_{kl}} \right) \end{array} \right\} \tag{S17}$$

S, T, and F are the overlap, the kinetic energy, and the intramolecular Fock matrixes, respectively. The formula means that we can evaluate E^{EXREP} using the LMOs of the isolated fragments.[9–11]

- **Polarization interaction term**

E^{POL} can be evaluated quantum mechanically. Although E^{POL} is smaller than E^{ES}, it should not be ignored because it promotes specific orientations of the molecules. E^{POL} is the interaction between the molecular fragments with static multipoles and the induced dipoles. The formula is given as Eq. S18.

$$E^{\text{POL}} = -\frac{1}{2}\sum_{k}\sum_{a}^{x,y,z} \mu_a^k F_a^{\text{mult},k} \tag{S18}$$

μ_a^k and $F_a^{\text{mult},k}$ are the a components of the induced dipole and the external fields at the point k, respectively. The external fields are from the static multipoles and nuclei of the other fragments. The induced dipoles at each polarizability point are calculated as Eq. S19.

$$\mu_a^k = \sum_{b}^{x,y,z} \alpha_{ab}^k F_b^{\text{total},k} \tag{S19}$$

α_{ab}^k is the ab component of the polarizability tensor of the polarized fragment molecule that determines the magnitude and directionality of the induced dipoles. In the EFP method, the polarizability tensor is evaluated using the LMOs obtained for the target small fragment molecules.[12]

- **Charge-transfer interaction term**

E^{CT} can only be evaluated by quantum mechanics because the origin is overlaps among the occupied and virtual molecular orbitals on the interacting molecules. In the EFP method, the charge-transfer energy of the fragment molecule M_1, induced by fragment molecule M_2, is expanded by the occupied and virtual canonical orbitals of the fragment molecules (Eq. S20).

$$\begin{aligned} E_{\mathrm{M}_1(\mathrm{M}_2)}^{\text{CT}} = 2 \sum_{i}^{occ\mathrm{M}_1} \sum_{l}^{vir\mathrm{M}_2} & \frac{V_{il}^{\mathrm{M}_2} - \sum_k^{all\mathrm{M}_1} S_{lk} V_{ik}^{\mathrm{M}_2}}{\left(1 - \sum_k^{all\mathrm{M}_1} {S_{lk}}^2\right)\left(F_{ii}^{\mathrm{M}_1} - T_{ll}\right)} \\ & \times \left\{ V_{il}^{\mathrm{M}_2} - \sum_{k}^{all\mathrm{M}_1} S_{lk} V_{ik}^{\mathrm{M}_2} + \sum_{j}^{occ\mathrm{M}_2} S_{ij} \left(T_{lj} - \sum_{k}^{all\mathrm{M}_1} S_{lk} T_{kj} \right) \right\} \end{aligned} \tag{S20}$$

S, T, F, and V^{M_2} are the overlap, the kinetic energy, the intramolecular Fock matrixes, and the electrostatic potential of molecule M_2 respectively.

- **Dispersion interaction term**

Focusing only on the isotropic interaction, E^{DISP} can be expressed by the following equation (Eq. S21).

$$E^{\mathrm{DISP}} = \sum_{\mathrm{M}_1,\mathrm{M}_2} \sum_{k\in\mathrm{M}_1} \sum_{l\in\mathrm{M}_2} \left(\frac{C_6^{kl}}{{R_{kl}}^6} + \frac{C_8^{kl}}{{R_{kl}}^8} + \frac{C_{10}^{kl}}{{R_{kl}}^{10}} + \cdots \right) \tag{S21}$$

$C_n(n = 6, 8, 10, \cdots)$ are the isotropic dispersion coefficients. The $1/R^6$ term is the van der Waals interaction term. Approximating the higher-order contribution as 1/3 of the van der Waals term (13), we obtain Eq. S22.

$$E^{\mathrm{DISP}} \simeq \frac{4}{3} \sum_{\mathrm{M}_1,\mathrm{M}_2} \sum_{k\in\mathrm{M}_1} \sum_{l\in\mathrm{M}_2} \frac{C_6^{kl}}{{R_{kl}}^6} \tag{S22}$$

The C_6 coefficients can be numerically evaluated using the 12-point Gauss-Legendre quadrature formula, and the dynamic polarizability tensor required for the evaluation can be calculated using the LMOs obtained for the target small fragment molecules.

Supplementary Figures and Tables

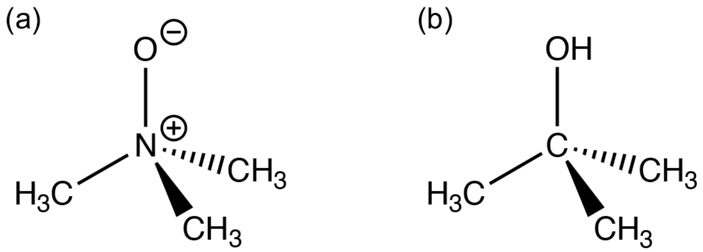

Figure S1. Chemical structures of (a) TMAO and (b) TBA. In this paper, the top and bottom of each compound are defined as depicted.

Table S1. Dipole moments (in units of D) evaluated with *ab initio* quantum chemistry calculations (MP2, CCSD), EFP, and classical force fields.

	**MP2**	**CCSD**	**EFP**	**Classical FF**
TMAO	5.05	5.03	5.17	5.53[14]
TBA	1.63	1.64	1.74	2.19[14]
H_2O	1.86	1.87	1.99	2.35[15]

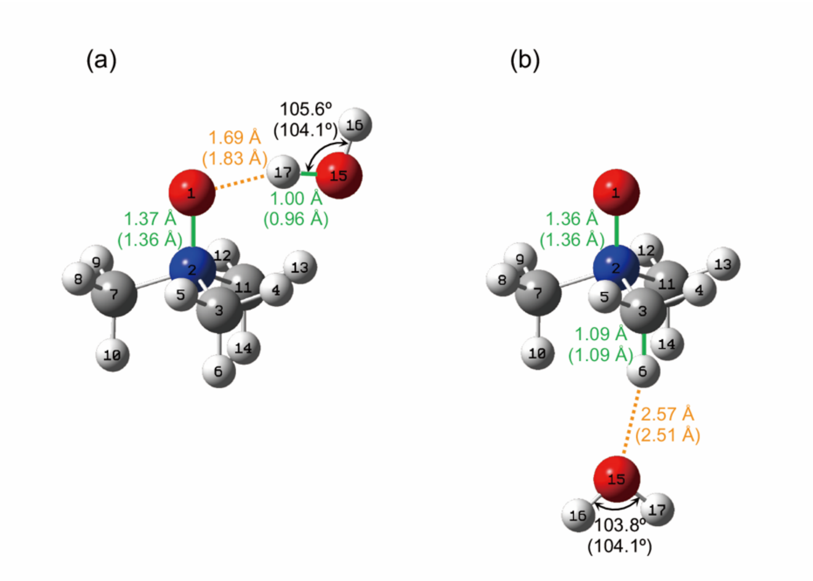

Figure S2. Optimized structures of TMAO–H_2O dimer models. A water molecule interacts with the top and bottom of TMAO in models (a) and (b). The values inside and outside the parentheses were obtained by EFP and MP2/aug-cc-pVTZ, respectively. The optimized *xyz* coordinates are listed in Table S2.

Table S2. Optimized *xyz* coordinates of TMAO–H_2O dimer models shown in Figure S2.

(a)

	MP2				EFP		
	x	y	z		x	y	z
O	0.203870	–0.000084	–1.168610	O	0.168501	–0.016039	–1.178783
N	–0.570475	–0.000008	–0.032690	N	–0.581535	–0.001255	–0.042755
C	–0.277775	1.216648	0.773581	C	–0.268780	1.221192	0.743537
H	0.777072	1.185649	1.029562	H	0.794330	1.189254	0.958701
H	–0.482091	2.070367	0.135255	H	–0.486743	2.068394	0.101462
H	–0.904497	1.230483	1.664866	H	–0.859468	1.253674	1.659583
C	–1.996131	–0.000030	–0.441257	C	–2.024764	–0.003198	–0.399877
H	–2.151468	0.887910	–1.045343	H	–2.195270	0.877093	–1.011050
H	–2.151421	–0.887973	–1.045349	H	–2.198128	–0.897291	–0.989846
H	–2.639867	–0.000041	0.437995	H	–2.645236	0.008515	0.496775
C	–0.277806	–1.216544	0.773761	C	–0.272688	–1.205565	0.772537
H	–0.482240	–2.070369	0.135614	H	–0.493403	–2.067162	0.150882
H	0.777061	–1.185561	1.029659	H	0.790528	–1.171917	0.986917
H	–0.904460	–1.230178	1.665099	H	–0.863442	–1.214245	1.689074
O	2.587196	0.000098	0.011615	O	2.618154	0.017427	0.015725
H	3.347385	–0.000760	–0.575152	H	3.418394	–0.120525	–0.498474
H	1.789590	–0.000027	–0.583923	H	1.913334	–0.002681	–0.637452

(b)

	MP2				EFP		
	x	y	z		x	y	z
O	–2.182053	–0.010263	0.044370	O	–2.169105	–0.015494	0.060935
N	–0.820784	–0.000985	0.004663	N	–0.808954	–0.001277	0.005079
C	–0.275189	–0.079853	1.386176	C	–0.254101	–0.071187	1.382649
H	–0.674146	0.773892	1.924847	H	–0.647837	0.785074	1.920522
H	–0.663572	–0.996043	1.819736	H	–0.633250	–0.986791	1.824728
H	0.814088	–0.071774	1.363937	H	0.836111	–0.061075	1.361630
C	–0.332122	–1.164866	–0.781272	C	–0.331418	–1.170192	–0.779807
H	–0.709958	–2.054312	–0.287076	H	–0.708477	–2.056094	–0.279286
H	–0.775913	–1.080172	–1.768162	H	–0.779596	–1.087794	–1.764626
H	0.756513	–1.158072	–0.828268	H	0.757483	–1.178721	–0.837505
C	–0.344734	1.252594	–0.637805	C	–0.351368	1.253121	–0.648792
H	–0.787178	1.280262	–1.628530	H	–0.799006	1.270027	–1.637152
H	–0.731937	2.073515	–0.042622	H	–0.742474	2.073591	–0.056017
H	0.743961	1.263168	–0.683489	H	0.737195	1.285695	–0.704268
O	2.975833	0.001622	–0.015012	O	2.943482	0.002452	–0.007104
H	3.564667	–0.758475	0.029958	H	3.530580	–0.758555	–0.007381
H	3.571004	0.756780	0.029568	H	3.538247	0.757471	–0.011134

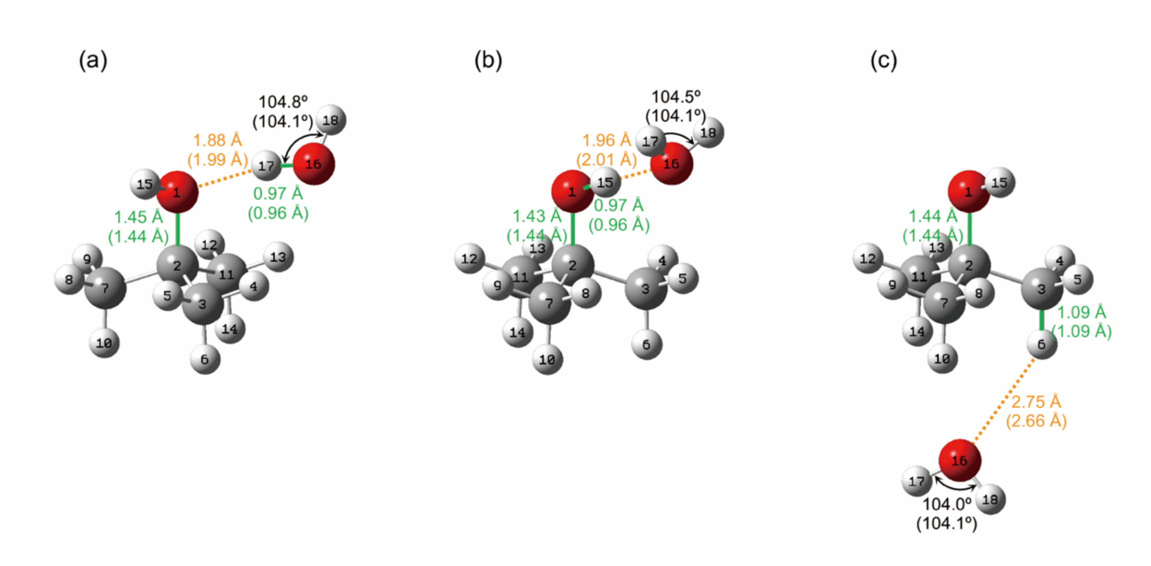

Figure S3. Optimized structures of TBA–H_2O dimer models. A water molecule interacts with the top and bottom of TBA in models (a), (b), and (c). The values inside and outside the parentheses were obtained by EFP and MP2/aug-cc-pVTZ, respectively. The optimized *xyz* coordinates are listed in Table S3.

Table S3. Optimized *xyz* coordinates of TBA–H_2O dimer models shown in Figure S3.

(a)

	MP2				EFP		
	x	y	z		x	y	z
O	0.228591	–0.028786	1.127264	O	0.145970	–0.011754	1.188289
C	–0.660023	0.008809	–0.017792	C	–0.650941	0.007241	–0.008033
C	–0.346999	–1.162988	–0.937027	C	–0.295753	–1.189145	–0.880618
H	0.695851	–1.128945	–1.250156	H	0.768360	–1.178022	–1.113407
H	–0.528688	–2.110034	–0.424528	H	–0.528293	–2.121996	–0.362730
H	–0.983337	–1.131880	–1.821902	H	–0.861272	–1.171026	–1.812861
C	–2.098390	–0.044656	0.472315	C	–2.128714	–0.012051	0.359014
H	–2.290546	–0.982223	0.998285	H	–2.379093	–0.934431	0.887235
H	–2.294952	0.783361	1.152315	H	–2.363130	0.832373	1.006050
H	–2.790577	0.017172	–0.367674	H	–2.751270	0.044422	–0.534544
C	–0.362379	1.331186	–0.699730	C	–0.283460	1.307445	–0.699185
H	–0.546806	2.156177	–0.012310	H	–0.511436	2.151921	–0.049892
H	0.678003	1.363208	–1.020857	H	0.782110	1.321101	–0.925153
H	–1.003599	1.451515	–1.572414	H	–0.842808	1.415233	–1.628159
H	0.051896	–0.849464	1.604554	H	–0.057689	–0.827368	1.661594
O	2.815428	–0.070078	–0.023046	O	2.768905	–0.054056	–0.036495
H	1.998351	–0.000528	0.500056	H	2.044547	0.036493	0.588744
H	3.469003	0.428431	0.474288	H	3.534184	0.276842	0.441699

(b)

	MP2				EFP		
	x	y	z		x	y	z
O	0.195784	–0.001755	–1.109987	O	0.165273	–0.002666	–1.132766
C	–0.712373	0.000037	–0.002674	C	–0.716375	0.000058	0.002709
C	–0.492779	–1.247834	0.845084	C	–0.470309	–1.243270	0.846800
H	–0.622243	–2.139596	0.232177	H	–0.607408	–2.138259	0.241119
H	0.518090	–1.249746	1.256680	H	0.548522	–1.242545	1.239835
H	–1.199895	–1.285243	1.674998	H	–1.157885	–1.280533	1.692417
C	–0.499649	1.255612	0.835362	C	–0.492443	1.262914	0.823660
H	0.511223	1.266312	1.246825	H	0.525653	1.286720	1.217877
H	–0.634017	2.141884	0.215598	H	–0.643655	2.143924	0.201074
H	–1.206914	1.295510	1.665030	H	–1.181854	1.304483	1.667577
C	–2.099655	–0.006182	–0.618795	C	–2.113407	–0.017708	–0.589952
H	–2.231845	0.875455	–1.245340	H	–2.262858	0.859697	–1.218058
H	–2.226884	–0.893264	–1.238652	H	–2.247454	–0.909226	–1.201438
H	–2.864446	–0.005404	0.158001	H	–2.862097	–0.016789	0.201846
H	1.094899	0.003091	–0.746164	H	1.070484	0.008395	–0.799313
O	2.908835	0.000519	–0.012800	O	2.920178	0.001260	–0.013751
H	3.433925	0.756689	–0.294045	H	3.458468	0.749614	–0.285827
H	3.417888	–0.765600	–0.296672	H	3.431680	–0.766198	–0.284284

(c)	MP2				EFP		
	x	y	z		x	y	z
O	2.208348	0.090799	0.027447	O	2.185127	0.077314	0.018392
C	0.771784	0.007751	–0.000563	C	0.749396	0.007336	–0.001451
C	0.334572	–1.071778	–0.981141	C	0.301958	–1.088906	–0.959029
H	0.742426	–0.864122	–1.970023	H	0.703088	–0.901205	–1.954275
H	0.694404	–2.050617	–0.656068	H	0.659521	–2.062878	–0.618412
H	–0.753152	–1.107210	–1.042793	H	–0.786068	–1.131336	–1.019161
C	0.243130	–0.291152	1.395380	C	0.228771	–0.258517	1.404672
H	0.610289	–1.260272	1.741264	H	0.584252	–1.225324	1.767175
H	0.581189	0.474647	2.093173	H	0.579438	0.516823	2.084774
H	–0.846449	–0.319095	1.389750	H	–0.861551	–0.273408	1.418412
C	0.308366	1.374190	–0.470184	C	0.300621	1.370778	–0.494289
H	0.639850	2.141730	0.228714	H	0.656748	2.147122	0.181845
H	0.729711	1.592867	–1.451005	H	0.708046	1.560993	–1.486515
H	–0.778602	1.392568	–0.537879	H	–0.786939	1.417706	–0.544215
H	2.540706	–0.761783	0.332751	H	2.514518	–0.774961	0.327988
O	–3.060385	–0.020661	–0.013552	O	–2.976344	–0.021826	–0.002606
H	–3.547665	0.776439	0.216740	H	–3.470761	0.788430	0.148560
H	–3.743526	–0.690313	–0.116732	H	–3.655039	–0.690017	–0.131873

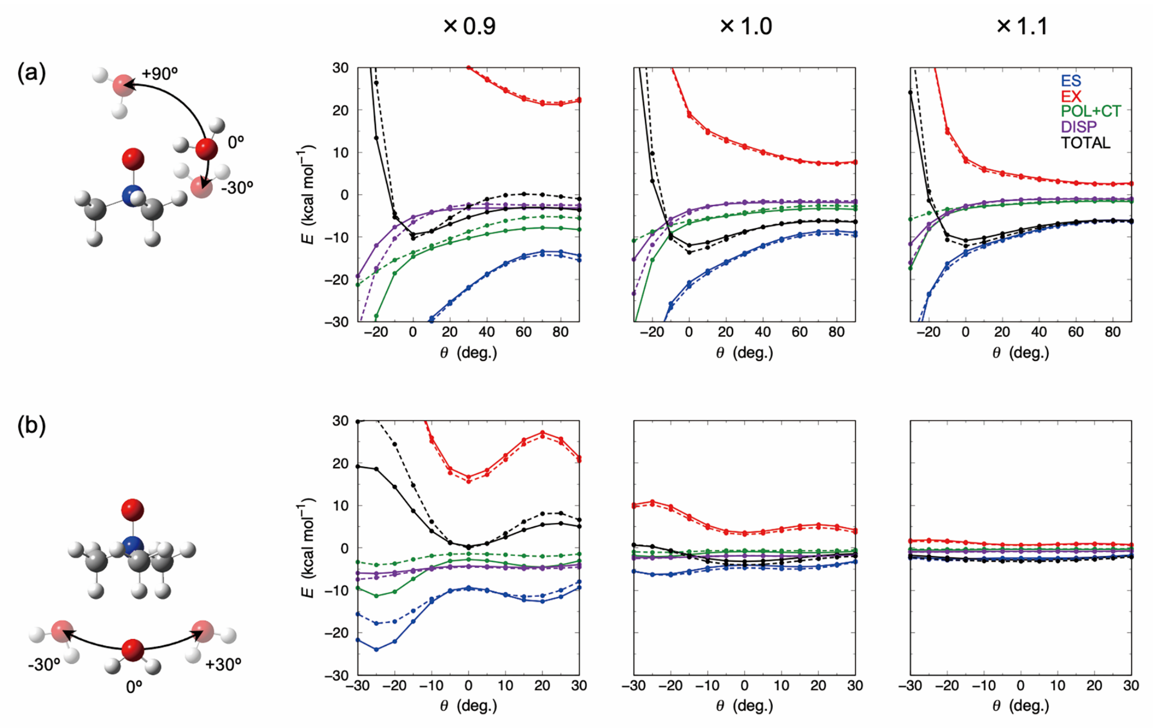

Figure S4. Potential energy surfaces (solid: MP2/aug-cc-pVTZ vs dashed: EFP) for TMAO–H_2O dimer models. Optimized O_{TMAO}–O_{water} distances varied by a factor of 0.9 to 1.1. LMO-EDA was applied to the MP2 results to obtain each interaction component.

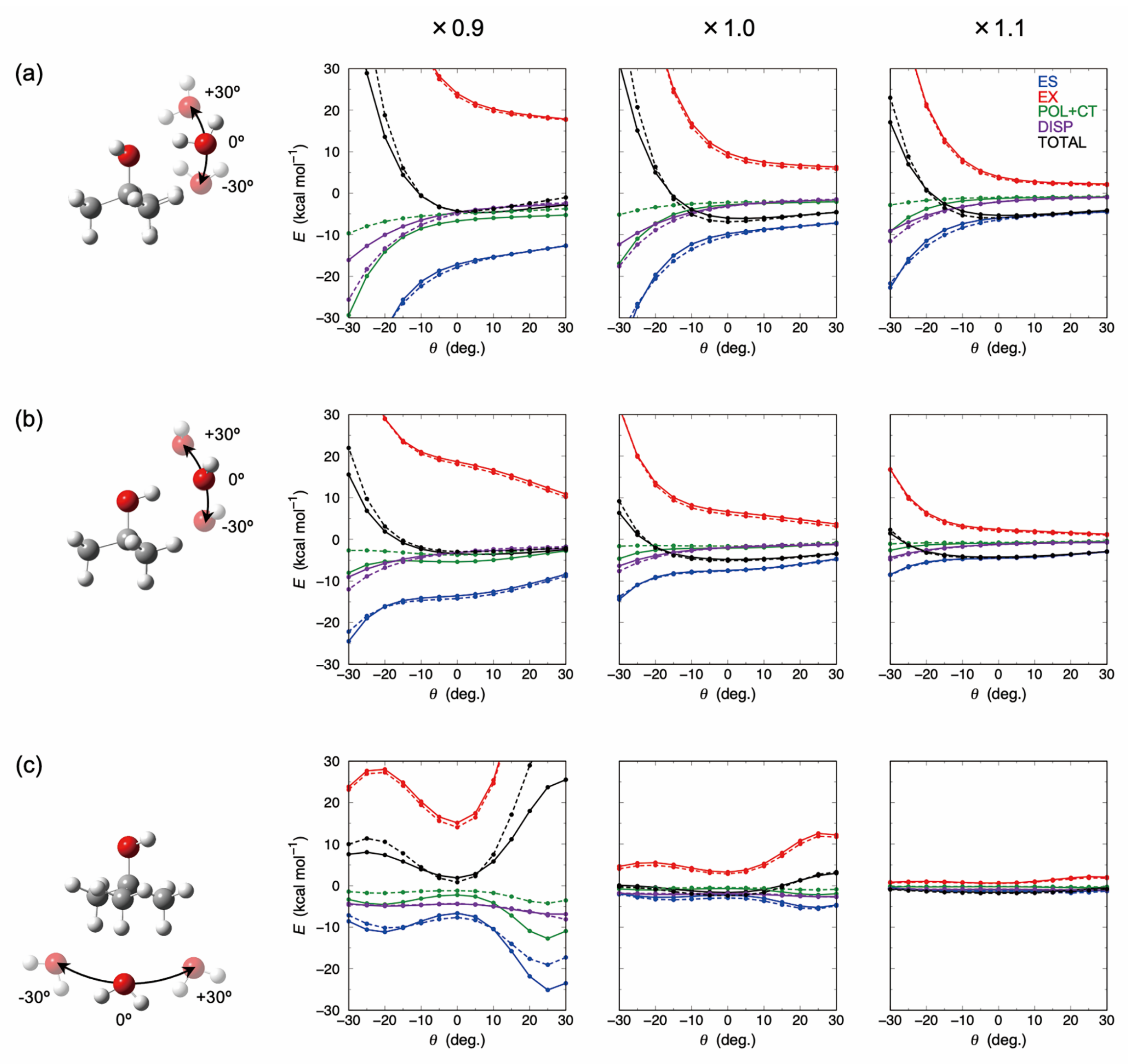

Figure S5. Potential energy surfaces (solid: MP2/aug-cc-pVTZ vs dashed: EFP) for TBA–H_2O dimer models. Optimized O_{TBA}–O_{water} distances varied by a factor of 0.9 to 1.1. LMO-EDA was applied to the MP2 results to obtain each interaction component.

Table S4. Diffusion coefficients (in units of 10^{-9} m^2 s^{-1}) evaluated with the EFP-MD simulations.

	D_{water}	D_{solute}
TMAO aq.	2.2 ± 0.0	0.5 ± 0.1
TBA aq.	2.3 ± 0.0	0.6 ± 0.1
Pure water	2.4 ± 0.1	–

Experimental results are summarized in Refs.16 and 17.

Table S5. Average coordination numbers of water around the top (hydrophilic N^+O^-/OH groups) and bottom (hydrophobic CH_3– groups) of TMAO/TBA.

	**Top**		**Bottom**
	$\mathbf{O_{TMAO/TBA}}$–$\mathbf{O_{water}}$	$\mathbf{O_{TMAO/TBA}}$–$\mathbf{H_{water}}$	$\mathbf{X_{TMAO/TBA}}$–$\mathbf{O_{water}}$
TMAO aq.	3.3 (r < 3.4 Å)	3.3 (r < 2.6 Å)	0.7 (r < 2.7 Å)
TBA aq.	3.3 (r < 3.5 Å)	2.0 (r < 2.6 Å)	0.3 (r < 2.7 Å)

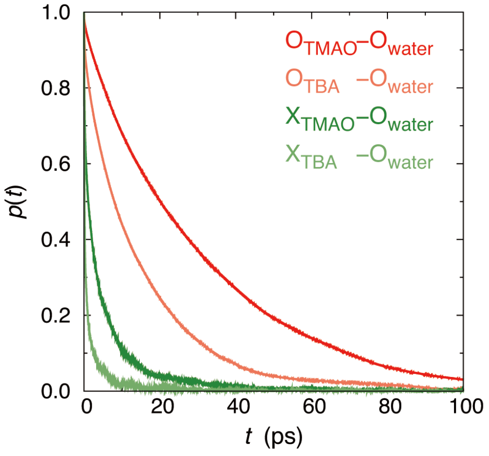

Figure S6. The TMAO/TBA···H_2O interaction correlation functions within the first hydration shell of dilute aqueous TMAO/TBA solutions. $X_{TMAO/TBA}$ was defined as the center of mass of the three axial hydrogen atoms of the CH_3– groups in TMAO/TBA.

Table S6. The TMAO/TBA···H_2O interaction lifetimes (ps) within the first hydration shell around the top (hydrophilic N^+O^-/OH groups) and bottom (hydrophobic CH_3– groups) of TMAO/TBA.

	**Top**	**Bottom**
	**$O_{TMAO/TBA}$–O_{water}**	**$X_{TMAO/TBA}$–O_{water}**
TMAO aq.	31.2 (95%)	6.9 (52%)
	0.6 (5%)	0.4 (48%)
TBA aq.	16.5 (82%)	–
	0.8 (18%)	–

Table S7. Average dipole moments (in units of D) of water and TMAO/TBA molecules in the dilute aqueous solutions. The water molecules, those around the top (hydrophilic $N^{+}O^{-}$/OH groups) and bottom (hydrophobic CH_3– groups) of TMAO/TBA are analyzed separately. The data (before averaging) are illustrated in Figure 3.

	**TMAO aq.**			**TBA aq.**		
Solute	9.39 ± 0.50			3.23 ± 0.39		
Water	r < 3.5 Å	r < 4.0 Å	r < 4.5 Å	r < 3.5 Å	r < 4.0 Å	r < 4.5 Å
Top	3.22 ± 0.28	3.03 ± 0.23	2.85 ± 0.20	3.01 ± 0.20	2.92 ± 0.19	2.84 ± 0.20
Bottom	2.84 ± 0.16	2.80 ± 0.19	2.83 ± 0.20	2.79 ± 0.16	2.80 ± 0.19	2.84 ± 0.20

Average dipole moments of water in the bulk: 2.87 D.

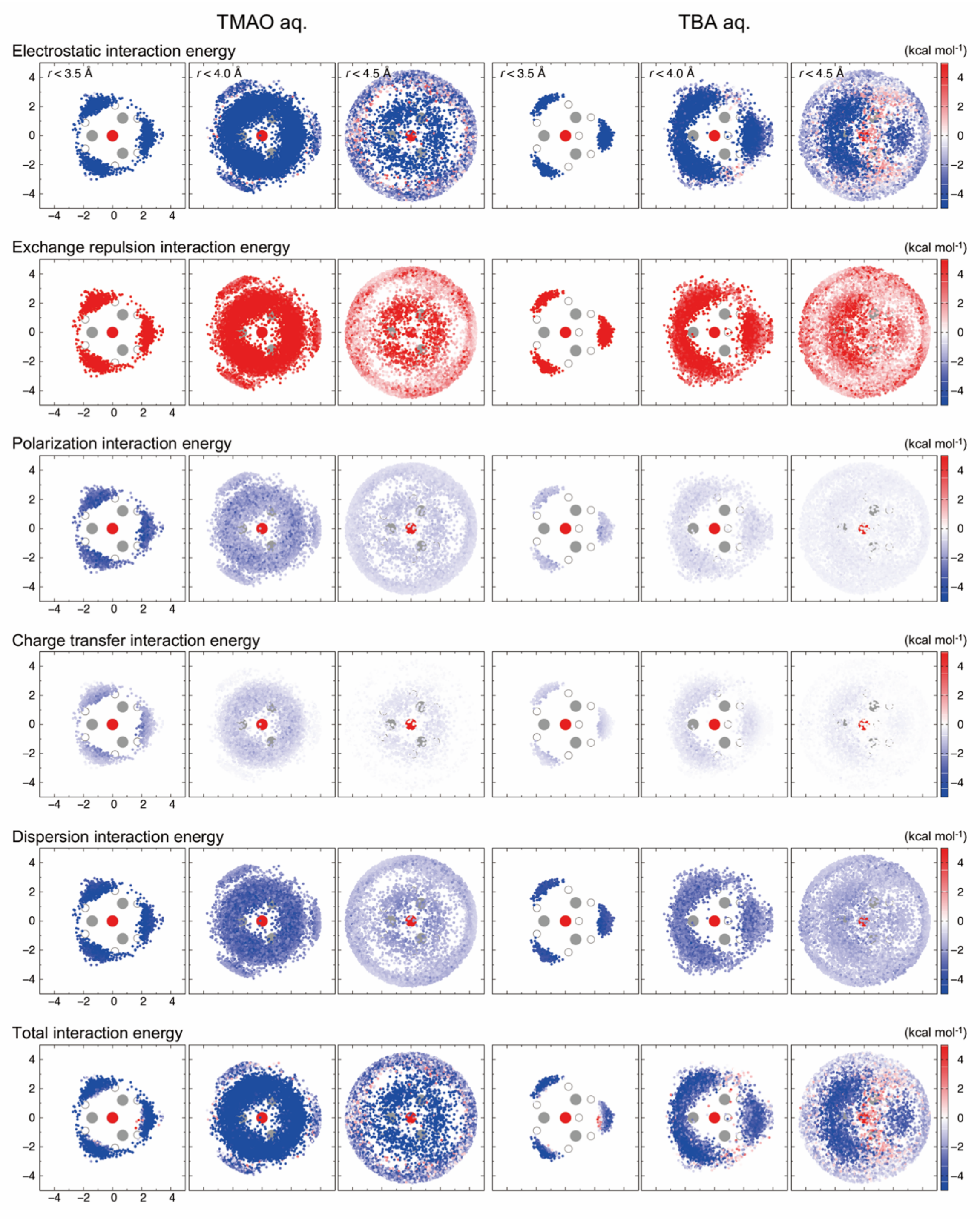

Figure S7. 1 ns water fluctuation colored by the TMAO/TBA–water interactions. The water molecules within 3.5, 4.0, and 4.5 Å from the solute are observed from the top.

Table S8. Average intermolecular interaction energy (kcal mol^{-1}) around the top (hydrophilic $N^{+}O^{-}$/OH groups) of TMAO/TBA. The data (before averaging) are illustrated in Figure S7.

	**TMAO aq.**			**TBA aq.**		
	r < 3.5 Å	r < 4.0 Å	r < 4.5 Å	r < 3.5 Å	r < 4.0 Å	r < 4.5 Å
ES	–20.1 ± 4.4	–12.6 ± 4.4	–2.9 ± 3.1	–10.3 ± 2.3	–7.0 ± 2.5	–2.1 ± 2.3
EX	21.3 ± 8.0	11.1 ± 5.3	2.2 ± 1.5	12.9 ± 4.1	7.0 ± 3.1	2.2 ± 1.5
POL	–3.4 ± 0.8	–1.8 ± 0.6	–0.8 ± 0.2	–1.2 ± 0.4	–0.7 ± 0.2	–0.3 ± 0.1
CT	–1.5 ± 0.5	–0.9 ± 0.4	–0.1 ± 0.2	–0.7 ± 0.2	–0.5 ± 0.2	–0.1 ± 0.1
DISP	–4.9 ± 0.9	–3.1 ± 0.8	–1.3 ± 0.4	–4.0 ± 0.6	–2.6 ± 0.5	–1.4 ± 0.4
TOTAL	–8.7 ± 3.0	–7.4 ± 2.4	–2.9 ± 2.6	–3.3 ± 1.6	–3.7 ± 1.4	–1.6 ± 1.8

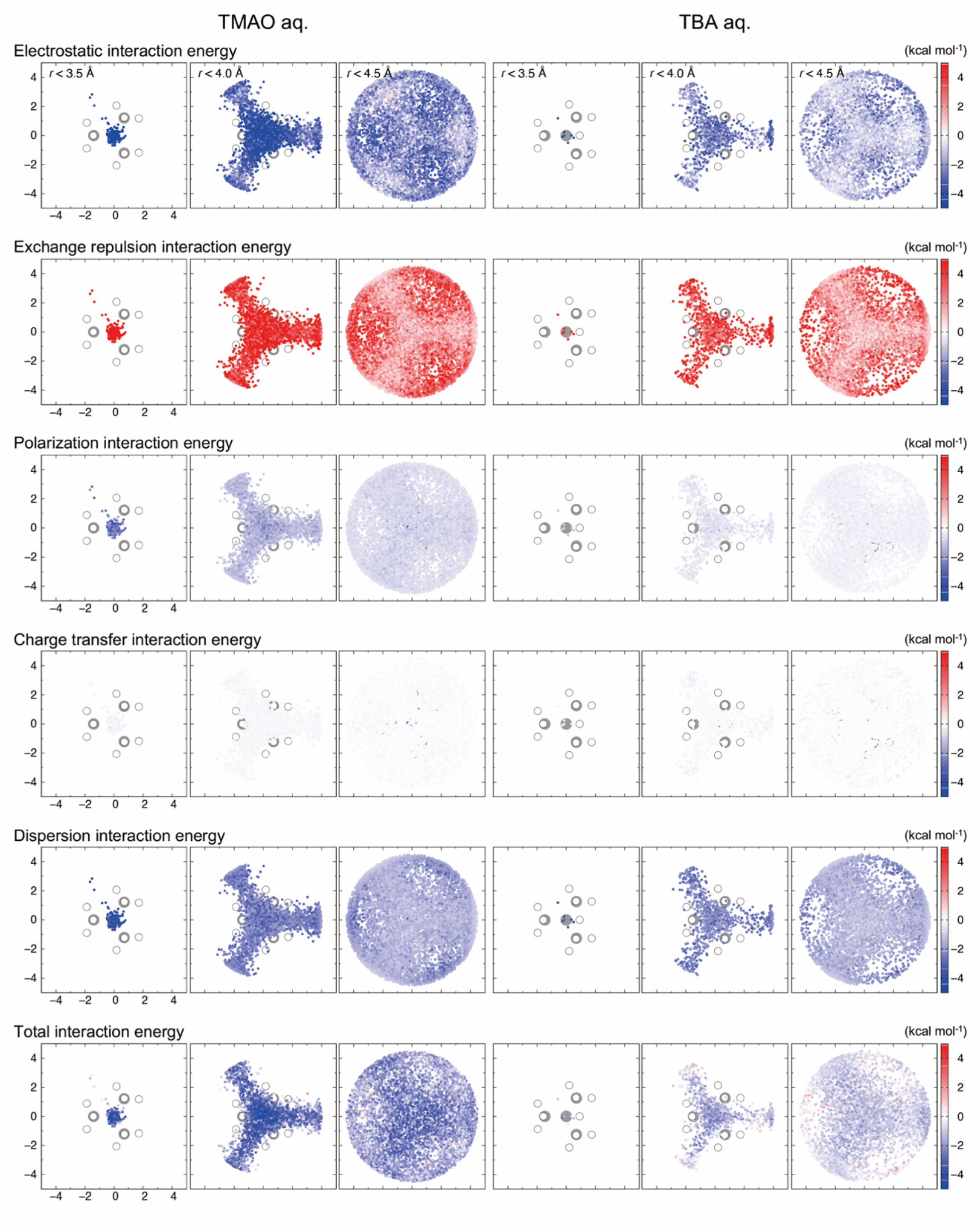

Figure S8. 1 ns water fluctuation colored by the TMAO/TBA–water interactions. The water molecules within 3.5, 4.0, and 4.5 Å from the solute are observed from the bottom.

Table S9. Average intermolecular interaction energy (kcal mol^{-1}) around the bottom (CH_3– groups) of TMAO/TBA. The data (before averaging) are illustrated in Figure S8.

	**TMAO aq.**			**TBA aq.**		
	$r < 3.5$ Å	$r < 4.0$ Å	$r < 4.5$ Å	$r < 3.5$ Å	$r < 4.0$ Å	$r < 4.5$ Å
ES	–7.1 ± 0.8	–3.9 ± 1.3	–1.8 ± 1.1	–5.1 ± 0.8	–2.5 ± 0.9	–1.2 ± 0.8
EX	10.6 ± 2.1	4.9 ± 1.9	2.3 ± 1.3	9.6 ± 2.2	4.3 ± 1.6	2.1 ± 1.3
POL	–2.7 ± 0.5	–1.4 ± 0.4	–0.7 ± 0.2	–1.1 ± 0.2	–0.5 ± 0.2	–0.3 ± 0.1
CT	–0.2 ± 0.0	–0.1 ± 0.1	0.0 ± 0.0	–0.2 ± 0.0	–0.1 ± 0.1	–0.1 ± 0.1
DISP	–4.1 ± 0.4	–2.5 ± 0.5	–1.5 ± 0.4	–4.1 ± 0.5	–2.4 ± 0.5	–1.5 ± 0.4
TOTAL	–3.5 ± 1.2	–3.0 ± 1.2	–1.8 ± 1.0	–0.8 ± 1.2	–1.2 ± 0.9	–0.8 ± 0.6

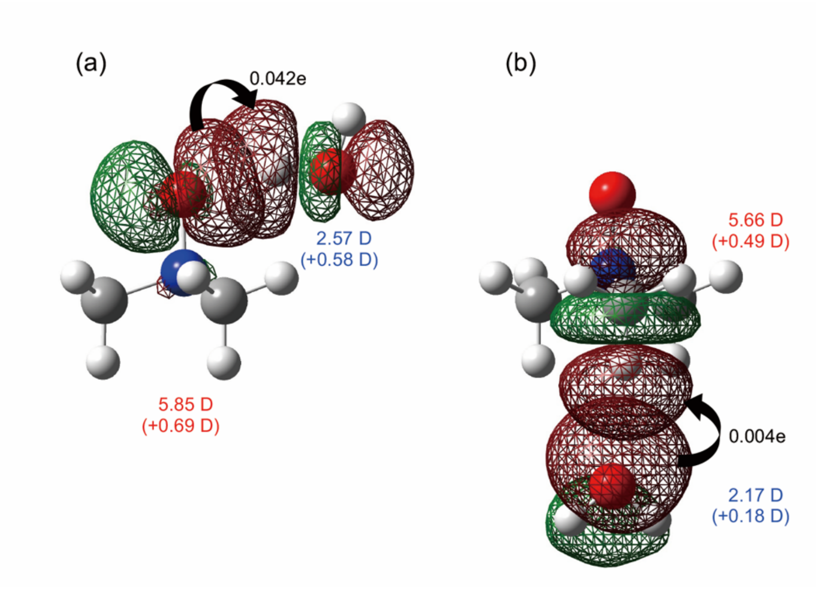

Figure S9. NBO analysis results for the optimized structures of the TMAO–H_2O dimer models in Figure S2. The black numerals are the charge-transfer amounts. The red and blue numerals are the dipole moments of TMAO and H_2O, respectively. The values inside the parentheses are the increments from the gas phase. The NBOs are depicted with an isovalue of 0.03 a.u.

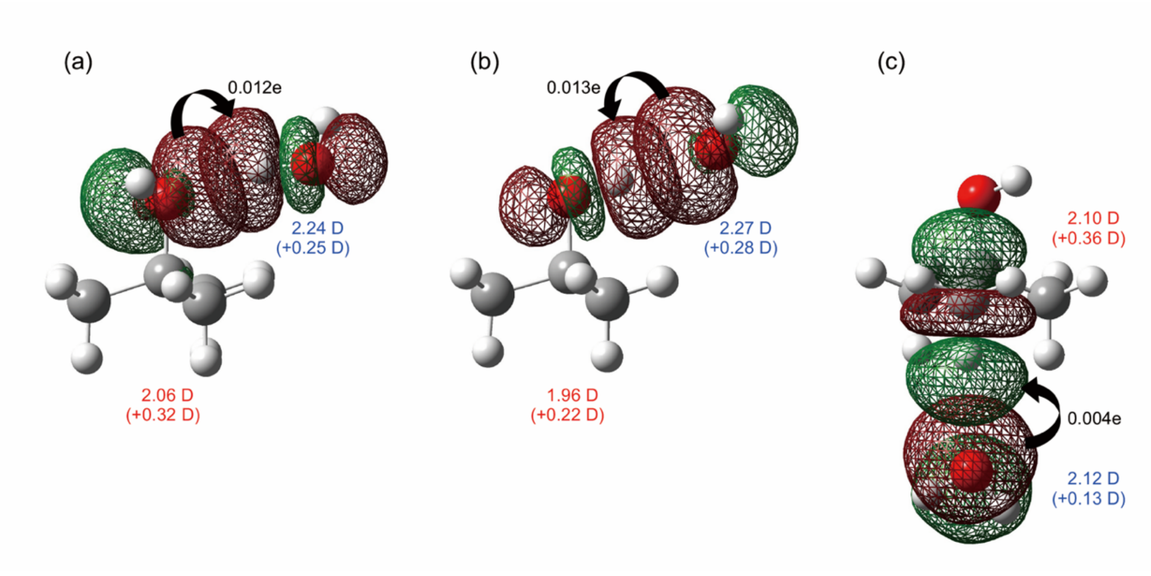

Figure S10. NBO analysis results for the optimized structures of the TBA–H_2O dimer models in Figure S3. The black numerals are the charge-transfer amounts. The red and blue numerals are the dipole moments of TBA and H_2O, respectively. The values inside the parentheses are the increments from the gas phase. The NBOs are depicted with an isovalue of 0.03 a.u.

Table S10. NBO second-order perturbation analysis results in the optimized structures of the TMAO/TBA–H_2O dimer models illustrated in Figures S9 and S10. $E_{\mathrm{DA}}^{(2)}$, $\varepsilon_{\mathrm{A}} - \varepsilon_{\mathrm{D}}$, and F_{DA} are the donor–acceptor stabilization energy, energy difference between acceptor and donor orbitals, and Fock matrix elements in the NBO basis, respectively.

		$E_{\mathrm{DA}}^{(2)}$ (kcal mol^{-1})	$\varepsilon_{\mathrm{A}} - \varepsilon_{\mathrm{D}}$ (a.u.)	F_{DA} (a.u.)
TMAO (see Figure S9)	(a)	28.48	1.20	0.166
	(b)	0.64	1.37	0.027
TBA (see Figure S10)	(a)	8.57	1.31	0.095
	(b)	7.75	1.51	0.097
	(c)	0.44	1.24	0.021

Supplementary References

1. Kuroki, N. & Mori, H. Effective fragment potential version 2 - molecular dynamics (EFP2-MD) simulation for investigating solution structures of ionic liquids. *Chem. Lett.* **45**, 1009–1011 (2016).
2. Kuroki, N. & Mori, H. Applicability of effective fragment potential version 2 - molecular dynamics (EFP2-MD) simulations for predicting excess properties of mixed solvents. *Chem. Phys. Lett.* **694**, 82–85 (2018).
3. Kuroki, N. & Mori, H. Applicability of effective fragment potential version 2-molecular dynamics (EFP2-MD) simulations for predicting dynamic liquid properties including the supercritical fluid phase. *J. Phys. Chem. B* **123**, 194–200 (2019).
4. Day, P. N., Jensen, J. H., Gordon, M. S. & Webb, S. P. An effective fragment method for modeling solvent effects in quantum mechanical calculations. *J. Chem. Phys.* **105**, 1968–1986 (1996).
5. Gordon, M. S. *et al.* The effective fragment potential method: A QM-based MM approach to modeling environmental effects in chemistry. *J. Phys. Chem. A* **105**, 293–307 (2001).
6. Gordon, M. S., Fedorov, D. G., Pruitt, S. R. & Slipchenko, L. V. Fragmentation methods: A route to accurate calculations on large systems. *Chem. Rev.* **112**, 632–672 (2012).
7. Stone, A. J. Distributed multipole analysis, or how to describe a molecular charge distribution. *Chem. Phys. Lett.* **83**, 233–239 (1981).
8. Ghosh, D. *et al.* Noncovalent interactions in extended systems described by the effective fragment potential method: Theory and application to nucleobase oligomers. *J. Phys. Chem. A* **114**, 12739–12754 (2010).
9. Jensen, J. H. & Gordon, M. S. An approximate formula for the intermolecular Pauli repulsion between closed shell molecules. *Mol. Phys.* **89**, 1313–1325 (1996).
10. Jensen, J. H. Modeling intermolecular exchange integrals between nonorthogonal molecular orbitals. *J. Chem. Phys.* **104**, 7795–7796 (1996).
11. Jensen, J. H. & Gordon, M. S. An approximate formula for the intermolecular Pauli repulsion between closed shell molecules. II. Application to the effective fragment potential method. *J. Chem. Phys.* **108**, 4772–4782 (1998).
12. Li, H., Netzloff, H. M. & Gordon, M. S. Gradients of the polarization energy in the effective fragment potential method. *J. Chem. Phys.* **125**, 194103 (2006).
13. Adamovic, I. & Gordon, M. S. Dynamic polarizability, dispersion coefficient C_6 and dispersion energy in the effective fragment potential method. *Mol. Phys.* **103**, 379–387 (2005).
14. Bandyopadhyay, D., Kamble, Y. & Choudhury, N. How different are the characteristics of aqueous solutions of *tert*-butyl alcohol and trimethylamine-N-oxide? A molecular dynamics simulation study. *J. Phys. Chem. B* **122**, 8220-8232 (2018).
15. Markthaler, D., Zeman, J., Baz, J., Smiatek, J. & Hansen, N. Validation of trimethylamine-N-oxide (TMAO) force fields based on thermophysical properties of aqueous TMAO solutions. *J. Phys. Chem. B* **121**, 10674–10688 (2017).
16. Sinibaldi, R. *et al.* The role of water coordination in binary mixtures. A study of two model amphiphilic molecules in aqueous solutions by molecular dynamics and NMR. *J. Phys. Chem. B* **110**, 8885–8892 (2006).
17. Paul, S. & Patey, G. N. Why *tert*-butyl alcohol associates in aqueous solution but trimethylamine-N-oxide does not. *J. Phys. Chem. B* **110**, 10514–10518 (2006).
